# Inequality in health service utilization among migrant and local children: a cross-sectional survey of children aged 0–14 years in Shenzhen, China

**DOI:** 10.1186/s12889-020-09781-4

**Published:** 2020-11-07

**Authors:** Xiatong Ke, Liang Zhang, Zhong Li, Wenxi Tang

**Affiliations:** 1 School of International Pharmaceutical Business, China Pharmaceutical University, Nanjing, Jiangsu 211198 PR China; 2 Center for Pharmacoeconomics and Outcomes Research of China Pharmaceutical University, Nanjing, Jiangsu 211198 PR China; 3 School of Medical and Health Management, Tongji Medical College of Huazhong University of Science and Technology, Wuhan, Hubei 430030 PR China; 4 Research Center for Rural Health Services, Hubei Province Key Research Institute of Humanities and Social Sciences, Wuhan, Hubei 430030 PR China

**Keywords:** Community-based healthcare, Service utilization, Inequality, Migrant child, Local child, Influencing factor

## Abstract

**Background:**

Shenzhen is characterized with the largest scale of migrant children among all the cities in China. Unequal access to health services among migrant and local children greatly affects health equity and has a profound impact on the quality of human capital. This study aimed to investigate differences in using community-based healthcare between local and migrant children and to identify the influencing factors in Futian District of Shenzhen.

**Methods:**

Households in 12 communities in Futian District of Shenzhen were randomly sampled. Children aged 0–14 years were investigated using self-administered questionnaire - the 2018 Survey of Health Service Needs of Chinese Residents. Differences in healthcare including physical examination, feeding guidance, development guidance, disease prevention guidance, injury prevention guidance, oral health guidance, and mental health guidance, were tested between local and migrant children. Binary logistic regressions were used in identifying potential influencing factors which affected the use in the above healthcare items.

**Results:**

A total of 936 participants from1512 families were sampled and 508 of them were included. Compared with local children, migrant children had less use of development guidance (OR = 0.417, 95% CI: 0.279–0.624) and oral health care guidance (OR = 0.557, 95% CI: 0.381–0.813). Children whose father received higher education level enjoyed a better use of disease prevention guidance as compared to whose father stopped at junior high school education or below (senior high vs junior high and below, OR = 1.286, 95% CI: 0.791–2.090; bachelor and above vs junior high and below, OR = 2.257, 95% CI: 1.417–3.595). Children whose fathers were blue-collar workers had less use of injury prevention guidance (OR = 0.750, 95% CI: 0.334–1.684) and mental health guidance (OR = 0.784, 95% CI: 0.295–2.080) as compared to whose father were white collar workers.

**Conclusions:**

Except feeding guidance, healthcare utilization were lower among migrant children than among local children. Generally, fathers have a stronger influence on children’s use of community-based healthcare than mothers do. The potential influence of fathers in promoting children’s healthcare use behaviors should be carefully considered, and fathers’ attention to children’s health should be increased.

## Introduction

### Background

With the rapid urbanization of China, many young and middle-aged people are migrating from rural areas to urban areas in search of better opportunities; at present, migrant workers account for about 36% of the total labor force in China [1]. According to the *Report on China’s Migrant Population Development 2016*, released by the Migrant Population Services and Management Division of National Population and Family Planning Commission, 60% of young and middle-aged migrants in China chose to migrate with their spouses and children in 2015. With the dominant migration pattern changed from individual migration to family migration, migrant children became an issue [2]. Migrant children, defined as children and adolescents aged 0–14 years who live temporarily with their migrant-worker parents/rural-to-urban migrant laborers or other guardians in their new cities of residence for more than half a year [3]. China remains an urban-rural separated household registration system till now, which has negative effects on migrant children’s equal rights in receiving same education and medical treatment as those children registered as urban households [4]. For example, migrant children are unable to go to public schools and enjoy quality education almost for free because public schools require locally registered permanent residence. Instead, many migrant children therefore are only able to attend private schools with expensive tuition which usually cost thousands of US dollars per year. Previous studies have also demonstrated that migrant children are a vulnerable group in terms of poor health, low quality of life [5, 6], and low utilization of basic healthcare services [7–9]. There is a significant gap between migrant children and local children in nutritional health [10], basic medical care [11–13], mental health [14], immunization [15, 16], and life satisfaction [17].

According to the “What Census Data Can Tell Us about Children in China: Facts and Figures 2015” United Nations Children’s Fund report, there were 34.26 million migrant children in China in 2015. Among them, Guangdong province has the largest number of 4.084 million migrant children aged 0–14, accounting for 12.0% in China, and among all the cities in Guangdong province, Shenzhen has the largest number of migrant children accounting for 22.3% of Guangdong [18]. According to the *Shenzhen Statistical Yearbook 2019*, there were 1.2 million migrant children in Shenzhen, namely there are 13 migrant children in every 100 children in the city. The main reason for the large number of migrant children in Guangdong and Shenzhen is that, at the early stage of reforms and opening-up in China dated back to 1978, the processing industry in the southern costal part of China boosted and therefore large population of migrant workers from central part of China has been attracted. And because Shenzhen locates directly connected to Hongkong, the biggest international trade port in Asia, it enjoyed the highest speed of gross domestic product per capita growth in China with the economy dominated mainly by labor-intensive industries [19].

“Healthy China 2030”, the top health strategy in China, pointed out the necessity of solving the health problems of key vulnerable groups such as women and children, migrant populations, and low-income groups. Therefore, reducing the health inequality between migrant children and local children are one of the top priorities of China’s health planning within next 10 years. Since 2009, following the functional framework of basic public health put forward by the World Health Organization in 1998 [20], China established a public health system aiming at providing equalized health care to urban and rural areas, different regions and various groups. During the past 10 years, the scope of this system has gradually been expanded [21]. At present, 11 types of basic public health services have been developed for urban and rural residents. The children healthcare programs are among the basic services, providing children with physical examination, feeding guidance, development guidance, disease prevention guidance, injury prevention guidance, oral health care guidance, and mental health guidance. The children healthcare programs had, to some extent, improved maternal and child health indicators and advanced equality in China [22]. But in recent years, the significant difference in services utilization between migrant children and local children has attracted the attention of researchers. These researches have mainly focused on the reasons of health inequality, and published studies have reported that family social and economic inequalities are the main sources of health inequality for children [23, 24]. Migrant children with economically disadvantaged families usually have worse health status than other children [25], especially in terms of mental health [10, 14], and planned immunization [26–28], mainly because socioeconomically vulnerable families do not have equal access to medical resources [29]. Therefore, the health inequality between migrant children and local children can be explained, to a large extent, by the socioeconomic status of these children’s families. In addition, parental occupation, education level, and marital status also have significant effects on children’s health [30]. There are few studies analyzed the utilization of healthcare programs of migrant children. Therefore, the objectives of this study is to investigate differences between migrant children and local children in the utilization of community-based healthcare services (including physical examination, feeding guidance, development guidance, disease prevention guidance, injury prevention guidance, oral health guidance, and mental health guidance) and to identify significant factors influencing these differences. And with the empirical evidences, we hope to propose suggestions to improve the utilization of healthcare services for migrant children.

## Methods

The study was part of the background research designed and implemented by the Research Center for Rural Health Services, Key Research Institute of Humanities & Social Sciences at Huazhong University of Science and Technology, aiming to understand the health service needs of Chinese residents and the factors influencing these needs. The research protocol was approved by the Ethics Committee of Tongji Medical College of Huazhong University of Science and Technology (IRB No. S459,2018). With the approval of this committee, written informed consent was obtained from the parents or legal guardians, and oral consent was obtained from the children.

### Setting

Shenzhen is established as one of -also the earliest - special economic zones (SEC) in 1980 in China, which were set up as the country’s economic reforms and opening pilots to the world. Futian District, the central urban area and transportation hub in Shenzhen, has the highest population density in the city [31]. The *Shenzhen Statistical Yearbook 2019* reported that, at the end of 2018, Futian District had an area of 78.66 km^2^ and a residential population of 1.6337 million, and 591,500 of whom were a “migrant population” not included in the household registration system. As of 2016, there were 10 streets (Yuanling, Nanyuan, Futian, Shatou, Meilin, Huafu, Xiangmihu, Lianhua, Huaqiangbei, and Fubao) and 94 communities in Futian District. In the survey developed in the present study, six streets with the largest administrative areas and highest concentrations of migrant population—Meilin Street, Shatou Street, Xiangmihu Street, Futian Street, Lianhua Street, and Huafu Street—were selected. Two communities were then randomly selected from each of these six streets and 120 households were randomly selected from each community for the administration of a questionnaire survey.

### Participants

Participants selected for this study were consistent with the definition of migrant children and adolescents in terms of age in the “Interim Measure of School Education for Temporary Migrant Children” which was jointly issued by the State Education Committee and the Public Security Department in 1998. Migrant children were defined as children aged 0–14 who were registered as living in other cities and who had lived with their migrant parents in Shenzhen for more than half a year without changing their household registration (indicating that, officially, no migration had occurred). Local children were defined as children aged 0–14 years whose city of registration was consistent with their place of residence, meaning that both were Shenzhen. The study therefore collected data on local children and migrant children living in selected households who were aged 0–14 years.

### Variables

#### Outcomes

Community-based healthcare developed in the Chinese basic public health system mainly focuses on preventive health care, aiming to protect and promote children’s physical and mental health and social adaptability and to provide comprehensive health care services appropriate for the growth and development characteristics of children of all ages. Depending on the children’s ages, this can be divided into 5 services: neonatal family visits, health management for newborns aged over 1 month, infant health management, health management for preschool children, and the management of health problems. What these 5 services have in common is that they all include 7 service items: physical examinations, feeding guidance, development guidance, disease prevention guidance, injury prevention guidance, oral health care guidance, and mental health guidance.

On the basis of the above 7 service items, some other service items are added according to the different ages of the children. For example, the services of neonatal family visits also include disease screening and vaccination; the service of infant health management also include blood test; the service of health management for preschool children also include traditional Chinese medicine health care and so on. Newborns can receive services provided by medical staff at home. Newborns aged over 1 month should go to community health services centers to receive services at the ages of 3,6,8,12,18,24,30,36 months. Children aged 4–6 years should attend the community health service center annually to receive corresponding services.

In this study, the dependent variable was whether a health services has been used. The health services included physical examination, feeding guidance, development guidance, disease prevention guidance, injury prevention guidance, oral health guidance, and mental health guidance. Take physical examination as an example, if the child has ever used the physical examination, the value of the dependent variable was coded as 1; otherwise, the dependent variable was coded as 0. Each dependent variable was examined by a separate model. Thus, there were seven regression models.

#### Influencing factors

**Fig. 1:**
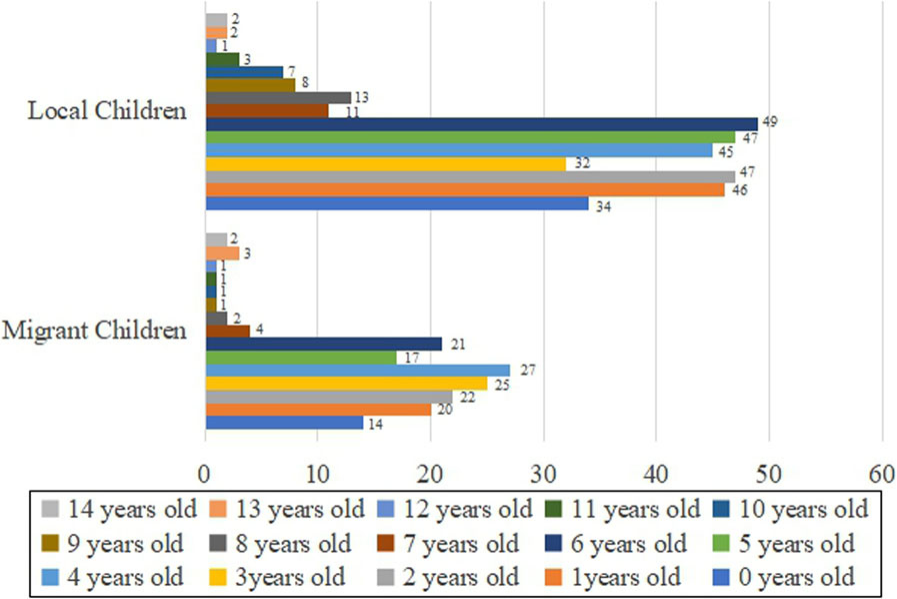
The age distribution of the local and migrant children

The influencing factors included in this study were the child’s sex, age, household registration status (local or migrant family), enrollment in medical insurance, annual family income, as well as both father’s and mother’s occupation, education level, and marital status. The coding of each of these categorical variables is shown in Table 1. The age is a continuous variable, and the age distribution of the local and migrant children is shown in Fig. 1.

**Table 1:** Coding of the independent variables

Variable	Assignment
Gender	Male = 1; female = 2
Household registration	Local child = 1; migrant children = 2
Family annual income	0 to 0.15 million yuan = 1; 0.15 to 0.5 million yuan = 2; 0.5 to 1 million yuan = 3; more than 1 million yuan = 4
Medical insurance of Child	Basic health insurance only =1; commercial insurance only =2; basic medical insurance + commercial insurance =3; no insurance or others = 4
Occupation of the parents	white-collar workers =1; blue-collar workers =2; Mixed white−/blue-collar workers =3; others = 4
Education level of the parents	Junior high school and below =1; Senior high school/ technical secondary school/ junior college = 2; Bachelor’s degree or above =3
Marital status of the parents	Unmarried =1; married = 2; divorced = 3; widowed = 4

For parental occupation, white-collar workers included staff members of the government or other institutions, clerks, and related personnel, whereas blue-collar workers included laborers in the industries of agriculture, forestry, animal husbandry, fishing, and water conservancy, as well as production and transportation equipment operators. Mixed white−/blue-collar workers included professional and technical personnel, commercial workers, and service personnel

In the main questionnaire there were other items such as geographical access to medical facilities, such as the type of medical institution located closest to the family, the distance between home and this medical institution, the type of transportation to the medical institution, and the time required to travel to the medical institution. However, these items were not used in the present study because the survey respondents lived in communities with similar levels of economic development, each community was equipped with a similar level of community health service centers, so there was no variation in geographical medical accessibility among these respondents.

### Quality control

Before the survey, community investigators who administered the questionnaire were trained, and unified coding rules for the questionnaire and implementation steps for the survey were explained in detail. Before investigation, quality-control staff members explained the precautions of filling out the questionnaire at the scene. For pre-school children (0–6 years of age), their parents or guardians completed the questionnaire on their behalf. Children at preliminary and junior high school (7–14 years of age) responded to the questionnaire themselves, with assistance from their parents or guardians. For data entry, Epidata 3.1 software was used by investigators and team members to create a database, and the accuracy of these data was checked to ensure the quality of the data input.

### Statistical methods

SPSS (Version 22.0) was used to organize and analyze the data. Descriptive statistics were used to analyze the general demographic characteristics of the children and their parents. The chi-square test was used to analyze differences between migrant children and local children in terms of the use of care. Binary logistic regression was used to analyze the specific factors influencing the difference in healthcare use between migrant children and local children. Forward (conditional) algorithm was developed, and the significance level was α = 0.05.

## Results

In this study, 4408 people in 1512 families from 12 communities were investigated. Data sources were collected on 936 children aged 0–14 years. Excluding incomplete questionnaires with missing responses, 508 valid questionnaires were included in the analysis, yielding an effective response rate of 54.27%.

### Demographic and socioeconomic characteristics

The general demographic and socioeconomic characteristics of the selected respondents are shown in Table 2. There were significant differences in annual family income, child’s enrollment in medical insurance, and parental occupation and educational level between migrant children and local children (all *Ps* < 0.05), but child’s gender, age or parental marital status did not differ significantly between migrant children and local children.

**Table 2:** Demographic characteristics of the surveyed children by migration status

Investigated factor	Local child (*n* = 347)	Migrate child (*n* = 161)	*χ*^2^	*P*-Value
**Gender**			0.470	0.493
Male	187 (53.89%)	92 (57.14%)		
Female	160 (46.11%)	69 (42.86%)		
**Annual family income**			41.619	< 0.001
Low (≤0.15 million yuan)	60 (17.29%)	63 (39.13%)		
Medium (0.15–0.5 million yuan)	211 (60.81%)	90 (55.90%)		
Medium to high (0.5–1 million yuan)	67 (19.31%)	6 (3.73%)		
High (1 million yuan or higher)	9 (2.59%)	2 (1.24%)		
**Child medical insurance participation**			38.084	< 0.001
Basic health insurance only	169 (48.70%)	80 (49.69%)		
Commercial insurance only	39 (11.24%)	8 (4.97%)		
Basic medical insurance + commercial insurance	101 (29.11%)	24 (14.91%)		
No insurance or others	38 (10.95%)	49 (30.43%)		
**Occupation of father**			36.437	< 0.001
White-collar workers	134 (38.62%)	28 (17.39%)		
Blue-collar workers	22 (6.34%)	6 (3.73%)		
Mixed white−/blue-collar workers	177 (51.01%)	104 (64.60%)		
Others	14 (4.03%)	23 (14.29%)		
**Occupation of mother**				
White-collar workers	166 (47.84%)	70 (43.48%)	12.162	0.007
Blue-collar workers	26 (7.49%)	6 (3.73%)		
Mixed white−/blue-collar workers	132 (38.04%)	60 (37.27%)		
Others	23 (6.63%)	25 (15.53%)		
**Education level of father**			100.268	< 0.001
Junior high school and below	40 (11.53%)	68 (42.24%)		
Senior high school/ technical secondary school/ junior college	102 (29.39%)	68 (42.24%)		
Bachelor’s degree or above	205 (59.08%)	25 (15.53%)		
**Education level of mother**			80.931	< 0.001
Junior high school and below	67 (19.31%)	83 (51.55%)		
Senior high school/ technical secondary school/ junior college	116 (33.43%)	61 (37.89%)		
Bachelor’s degree or above	164 (47.26%)	17 (10.56%)		
**Marital status of father**			1.075	0.783
Unmarried	13 (3.75%)	5 (3.11%)		
Married	330 (95.10%)	155 (96.27%)		
Divorced	2 (0.58%)	0 (0%)		
Widowed	2 (0.58%%)	1 (0.62%)		
**Marital status of mother**			2.305	0.512
Unmarried	4 (1.15%)	4 (2.48%)		
Married	332 (95.68%)	150 (43.23%)		
Divorced	5 (1.44%)	2 (1.24%)		
Widowed	6 (1.73%)	5 (3.11%)		

The annual family income of migrant children was generally lower than that of local children: The families of 17.29% of the local children and of 39.13% of the migrant children were in the low-income group. Migrant children’s insurance enrollment rate was significantly lower than that of local children, with 30.43% of migrant children not being enrolled in any health insurance at all. The percentage of children with fathers who performed mainly white-collar work was lower among migrant children (17.39%) than among local children (38.62%). The educational level of the parents of the migrant children was significantly lower than that of the parents of the local children: The educational level of the local children’s parents was mainly undergraduate or above, whereas the educational level of the migrant children’s parents was mainly junior high school or below.

### Use of community-based children healthcare

The differences in use of community-based healthcare for local and migrant children are shown in Table 3. Generally, the percentages of children who had received a physical examination, development guidance, disease prevention guidance, injury prevention guidance, oral health care guidance, and mental health guidance were significantly lower among migrant children than among local children, but there was no significant difference in the use of feeding guidance services.

**Table 3:** Differences between local children and migrant children in the utilization of health services

Basic child health management services	Overall (*n* = 508)	Household registration		*χ*^2^	*P* value
		Local children (*n* = 347)	Migrant children (*n* = 161)		
Physical examination	477 (93.9%)	333 (96.0%)	144 (89.4%)	8.170	0.004
Feeding guidance	369 (73.1%)	261 (75.2%)	108 (67.1%)	3.662	0.056
Development guidance	364 (71.7%)	269 (77.5%)	95 (59.0%)	18.562	< 0.001
Disease prevention guidance	273 (53.7%)	201 (57.9%)	72 (44.7%)	7.713	0.005
Injury prevention guidance	238 (46.9%)	178 (51.3%)	60 (37.3%)	8.693	0.003
Oral health guidance	265 (52.2%)	197 (56.8%)	68 (42.2%)	9.313	0.002
Mental health guidance	93 (18.1%)	72 (20.7%)	21 (13.0%)	4.366	0.037
None	9 (1.8%)	3 (0.9%)	6 (3.7%)	5.177	0.023
Unclear	11 (2.2%)	3 (0.9%)	8 (5.0%)	8.745	0.003

None means that the child has never used the basic child health management services; Unclear means that the child cannot clear remember clearly whether they have used the basic child health management services

### Factors influencing the children healthcare use

The variables listed in Table 4 were ultimately included in the final model for each binary logistic regression model. There are six models examining what factors have correlated with use of physical examination, development guidance, disease prevention guidance, injury prevention guidance, oral health care guidance, and mental health guidance.

**Table 4:** Binary logistic regression analysis of factors influencing the utilization of child health management services

Variable	*β*	Standard error	Wald test	Df	*P*-Value	Exp(*β*)	95% confidence interval
**Physical examination**							
Constant	1.931	0.245	61.857	1	< 0.001	6.895	–
Educational level of the mother (control = junior high school and below)							
Senior high school/ technical secondary school/ junior college	1.419	0.482	8.652	1	0.003	4.134	(1.606, 10.642)
Bachelor’s degree or above	1.442	0.482	8.941	1	0.003	4.230	(1.644, 10.888)
**Development guidance**							
Constant	1.238	0.129	92.6775	1	< 0.001	3.449	–
Household registration (control = local children) (*P* < 0.001)							
Migrant children	−0.874	0.205	18.085	1	< 0.001	0.417	(0.279,0.624)
**Disease prevention guidance**							
Constant	−0.298	0.195	2.353	1	0.125	0.742	–
Educational level of the father (control = junior high school and below) (*P* = 0.001)							
Senior high school/ technical secondary school/ junior college	0.251	0.248	1.029	1	0.310	1.286	(0.791,2.090)
Bachelor’s degree or above	0.814	0.238	11.739	1	0.001	2.257	(1.417,3.595)
**Injury prevention guidance**							
Constant	−0.088	0.242	0.132	1	0.716	0.916	–
Occupation of the father (control = White-collar workers) (*P* = 0.002)							
Blue-collar workers	−0.288	0.413	0.487	1	0.485	0.750	(0.334, 1.684)
Mixed white/blue-collar workers	−0.622	0.203	9.381	1	0.002	0.537	(0.361, 0.799)
Others	−1.240	0.418	8.808	1	0.003	0.289	(0.128, 0.656)
Annual family income (control = ≤0.15 million yuan) (*P* = 0.001)							
0.15–0.5 million yuan	0.476	0.227	4.415	1	0.036	1.610	(1.033, 2.511)
0.5–1 million yuan	0.485	0.308	2.474	1	0.116	1.624	(0.888, 2.970)
1 million yuan or higher	2.997	1.071	7.822	1	0.005	20.016	(2.451, 163.436)
**Oral health care guidance**							
Constant	0.273	0.108	6.327	1	0.012	1.313	–
Household registration (control = local children) (*P* = 0.002)							
Migrant children	−0.586	0.193	9.220	1	0.002	0.557	(0.381, 0.813)
**Mental health guidance**							
Constant	−1.341	0.218	37.953	1	< 0.001	0.262	–
Gender (control = male) (*P* = 0.009)							
Female	0.606	0.235	6.673	1	0.010	1.833	(1.157, 2.904)
Occupation of the father (control = White-collar workers) (*P* = 0.014)							
Blue-collar workers	−0.244	0.498	0.240	1	0.625	0.784	(0.295, 2.080)
Mixed white/blue-collar workers	−0.739	0.249	8.841	1	0.003	0.478	(0.293, 0.777)
Others	−1.050	0.562	3.495	1	0.602	0.350	(0.116, 1.052)

The results showed that the most important factor influencing the utilization of physical examination services was mother’s educational level. Compared with children whose mothers had a junior high school education or lower, the odds of having used physical examination services were 4.134 times (95% CI: 1.606, 10.642) higher for children whose mothers had a senior high school/technical secondary school/junior college education and 4.230 times (95% CI:1.644, 10.888) higher for children whose mothers had an undergraduate education or higher. The most important factor influencing the use of disease prevention guidance services was father’s educational level. The odds of having used prevention guidance services were 2.257 times (95% CI: 1.417, 3.595) higher for children whose father had an undergraduate education or higher than for children whose father had a junior high school education or lower. The factors influencing the utilization of injury prevention guidance services were father’s occupation and annual family income. In terms of father’s occupation, the odds of having used injury prevention guidance services were 46.3% lower (odds ratio [OR] = 0.537, 95% CI: 0.361, 0.799) for children whose fathers were mixed white−/blue-collar workers compared with children whose fathers were white-collar workers. As for the effect of annual family income, the odds of having used injury prevention guidance were 1.624 times higher (95% CI: 0.888, 2.970) in the middle-income group and 20.016 times (95% CI: 2.451, 163.436) higher in the high-income group, compared with the low-income group. The odds of having used mental health guidance services were 52.2% lower (OR = 0.478, 95% CI: 0.239, 0.777) for children whose fathers were mixed white−/blue-collar workers compared with children whose fathers were white-collar workers. In addition, the odds of having used mental health guidance services were 1.833 times higher (95% CI: 1.157, 2.904) for girls than for boys.

## Discussion

### Main findings

The uses of community-based children healthcare (except feeding guidance) were significantly lower among migrant children than among local children, indicating the presence of health inequality between local children and migrant children. This finding of health inequality between local children and migrant children is consistent with the results of previous studies. For example, in a study conducted in Italy comparing the oral health of children from a migrant population with that of local children, Ferrazzano et al. (2019) found that the decay-missing-filled teeth index, incidence of dental caries, and the unmet restorative treatment needs index were higher among the migrant population children than among other children [32]. And Zhang et al. (2019) also found that compared with urban counterparts, migrant children had more mental health problems with less use of community-based children healthcare services [14].

Compared with mothers, fathers have a stronger influence on children’s healthcare utilization. It is generally believed that mothers are primarily responsible for the care of children, and our study confirms that the use of physical examination services is higher among children whose mothers have higher education, which is consistent with the results of Ni et al. [33] and Wamani (2004), who found the most significant factor influencing the use of basic health services for children in Uganda was mother’s educational level [34]. However, our findings showed that father’s educational level and occupation actually had a higher impact on the use of children healthcare compared with these factors among mothers. For example, father’s education level affects the use of disease prevention guidance services, and children whose fathers have an undergraduate education or higher have better health status than do children whose fathers have less education. Father’s occupation and annual family income affect the use of injury prevention guidance services, which is especially high among children whose fathers are white-collar workers. We speculated that, because father’s educational level affects the socioeconomic status of the family which highly relates to health literacy in China, father’s educational level may serve to bridge the health gap between migrant children and local children. Concerning the Chinese culture specifically, China is a strictly patriarchal country. Father’s educational level and occupation largely determine the socioeconomic status of the family, fathers may have a greater impact than mothers on the utilization of healthcare for children. The results of the present study are consistent with Wang’s (2019) conclusion that father’s educational level can reduce health inequalities between migrant and non-migrant children and between urban and rural children, demonstrating that father’s educational level plays a significant role in regulating health equality [35].

### Implications

On the basis of the above findings, we believe that several measures can potentially improve the use of healthcare among migrant children. First, we still need to raise awareness of the following healthcare which have low use both in migrant and local children: disease prevention guidance, injury prevention guidance, oral health guidance, and psychological guidance to increase the awareness of these services. Second, migrant children differ substantially from local children in terms of their needs for development guidance, oral health care, and other services; therefore, doctors in community healthcare centers should organize targeted development guidance and oral health care lectures to enhance the awareness of the parents and children. Third, we should pay special attention to families with low educational level and mixed white−/blue-collar occupation type among the fathers of migrant children and formulate measures to attract the attention of these fathers, such as allowing them to take their children to community health service centers on a regular basis to receive healthcare. However, under the pressure of a big city like Shenzhen, most parents of migrant children still cannot spare enough time and flexible time with their children and help them accept their community-based healthcare services, leading to migrant children’s mistiming on the schedule of community-based healthcare services. Therefore, in order to better provide child health management services for migrant children, we should reduce the dependence of child service utilization on their parents. As the main provider of child health management services, the community-base health service centers need to take the initiative to provide children with corresponding services instead of waiting for parents to bring their children. Because the school is a place where children stay for a long time every day, the community-base health service center can cooperate with the school to improve the utilization of health services for migrant children.

### Limitations

This study had several limitations. The sample size is still small to fully represent the healthcare use status of migrant children in China. Although Shenzhen has the largest number of migrant children of all cities in China and the respondents for the study was randomly sampled, the results of the study may nevertheless have a certain bias. Second, the effective response rate is relatively low as compared to usual investigation. Because this study examined children aged 0–14 years, the respondents were not fully able to complete the questionnaire, which needed to be answered completely by their parents or with assistance. However, migrant parents are also busy workers with comparably insufficient education and even migrant parents are more easily to be disturbed by recall bias or hard to understand/ answer current investigation than local parents owing to different levels of education—a situation that may have affected the integrity and effectiveness of the questionnaire. Third, this study only included socioeconomic factors which is certainly not complete. Fourth, in terms of the depth of the study, this study only explored the use of children healthcare. The quality of these healthcare or health outcomes were not considered.

## Conclusions

This study discussed the inequality in community-based children healthcare between migrant children and local children in Futian District of Shenzhen, China. Fathers’ influence on children healthcare use was found to be more important than that of mothers, which implies that fathers should pay more attention and participate more actively in their children’s healthcare use and health education. With the labor migration pattern changing from individual migration to family migration, this study is of significance in improving the healthcare use and promoting the health of migrant children who are important to future human capital of a nation.

## Data Availability

The data that support the findings of this study are available from the Research Center for Rural Health Services, Key Research Institute of Humanities & Social Sciences at Huazhong University of Science and Technology but restrictions apply to the availability of these data, which were used under license for the current study, and so are not publicly available. Data are however available from the authors upon reasonable request and with permission of the Research Center for Rural Health Services, Key Research Institute of Humanities & Social Sciences at Huazhong University of Science and Technology.

## Abbreviations

CI: Confidence interval
OR: Odds ratio
Df: Degree of freedom
SSQ: Sum of Square
